# Introduction of a Simplified Psoriatic Arthritis Magnetic Resonance Imaging Score (sPsAMRIS): A Potential Tool for Treatment Monitoring in Peripheral Psoriatic Arthritis

**DOI:** 10.3390/diagnostics10121093

**Published:** 2020-12-15

**Authors:** Daniel B. Abrar, Christoph Schleich, Ralph Brinks, Christine Goertz, Miriam Frenken, Matthias Schneider, Sven Nebelung, Philipp Sewerin

**Affiliations:** 1 Department of Diagnostic and Interventional Radiology, Medical Faculty, University Dusseldorf, D-40225 Dusseldorf, Germany; Christoph.Schleich@med.uni-duesseldorf.de (C.S.); Miriam.Frenken@med.uni-duesseldorf.de (M.F.); Sven.Nebelung@med.uni-duesseldorf.de (S.N.); 2 Department and Hiller-Research-Unit for Rheumatology, UKD, Heinrich-Heine University Düsseldorf, D-40225 Dusseldorf, Germany; Ralph.Brinks@med.uni-duesseldorf.de (R.B.); christine.goertz@med.uni-duesseldorf.de (C.G.); matthias.schneider@med.uni-duesseldorf.de (M.S.); philipp.sewerin@med.uni-duesseldorf.de (P.S.)

**Keywords:** psoriatic arthritis, PsAMRIS, magnetic resonance imaging, OMERACT

## Abstract

Background: To evaluate whether a simplified (s) version of the psoriatic arthritis magnetic resonance imaging score (PsAMRIS), sPsAMRIS, is a potential tool for therapy monitoring in psoriatic arthritis (PsA). Methods: Seventeen patients with active psoriatic arthritis (PsA) underwent magnetic resonance imaging (MRI) at 3 T of the clinically dominant hand at baseline and after 6 months. Scoring was performed by two musculoskeletal radiologists in terms of the PsAMRIS and sPsAMRIS, which is a simplified version with reduced item numbers based on prior evaluation of responsiveness to change by standardized response means (SRMs). Both scores were compared by calculation of overall and each sub-score’s SRMs and relative efficacy (RE) after bootstrapping. Results: PsAMRIS sub-scores of MCP joints 3 and 4, and proximal interphalangeal (PIP) joint 4 had the highest SRM (−0.07 each), indicating highest responsiveness to change, and were, therefore, included in sPsAMRIS. Compared to PsAMRIS, sPsAMRIS was characterized by higher SRMs (sPsAMRIS: −0.13 vs. PsAMRIS: −0.02) and higher RE (29.46). sPsAMRIS and PsAMRIS were highly correlated at baseline (r = 0.75, *p* < 0.01 (Pearson’s correlation)) and at 6-month follow-up (r = 0.64, *p* = 0.01). Mean time burden for completion of scoring per MRI study was significantly reduced when using PsAMRIS (469 ± 87.03 s) as compared to sPsAMRIS (140.1 ± 21.25 s) (*p* < 0.001). Conclusion: Due to its similar responsiveness to change compared to standard PsAMRIS, and time efficiency, sPsAMRIS might be a potential diagnostic tool to quantitatively assess and monitor therapy in PsA.

## 1. Introduction

Psoriatic arthritis (PsA) is a chronic inflammatory disorder that results in progressive joint destruction if left untreated [1,2]. With a global prevalence of 0.05–0.25%, PsA constitutes one of the most common inflammatory joint diseases alongside rheumatoid arthritis (RA) and gout [3]. PsA may manifest in a variety of manifestations such as dactylitis, enthesitis, synovitis, or bone erosions [4]. Similar to RA, early diagnosis and targeted treatment of PsA are crucial for improved clinical outcomes, i.e., clinical remission or low disease activity [5]. Current treatment strategies suggest escalation of therapy in patients that do not demonstrate sufficient treatment response [6]; hence, early detection of treatment failure is of paramount importance. Consequently, there is a true clinical need for reliable tools for therapy monitoring in PsA.

Even though it is not part of the Classification Criteria for Psoriatic Arthritis (CASPAR) [7], magnetic resonance imaging (MRI) becomes increasingly important as a tool for early detection and monitoring of PsA-related joint involvement [5,8]. MRI is a reliable tool for detecting early PsA-related pathologies such as soft tissue swelling, enthesitis, bone marrow edema, and bone erosion [9,10]. In 2003, the Outcome Measures in Rheumatology Clinical Trials (OMERACT) working group presented a diagnostic scoring tool, the RA MRI Score (RAMRIS), for evaluation of changes related to RA, which has been validated used for outcome measurement ever since [11]. Subsequently, in 2007 the OMERACT Psoriatic Arthritis Magnetic Resonance Imaging Scoring System (PsAMRIS) was introduced [12]. PsAMRIS is a semi-quantitative scoring system that includes typical changes of peripheral PsA such as enthesitis, synovitis, tenosynovitis, periarticular inflammation, bone edema, bone erosion, and bone proliferation in 24 joints, resulting in a sum score [13]. PsAMRIS is an increasingly accepted diagnostic scoring tool for reliable and objective outcome measurements in controlled clinical trials investigating PsA [14]. A major drawback of PsAMRIS is the considerable time needed for scoring that may have prevented its more widespread implementation in clinical practice. 

The aim of this study was to systematically evaluate in a clinical cohort pf PsA which components of PsAMRIS are of superordinate diagnostic relevance to be included in its simplified version, termed sPsAMRIS. Hence, our hypothesis was that sPsAMRIS is as diagnostically reliable as PsAMRIS, while reducing the time burden and complexity associated with standard scoring procedures of PsAMRIS. 

## 2. Materials and Methods

### 2.1. Patients

Seventeen patients with PsA (mean age, 53.7 ± 11.6 years; range, 26–72 years; male/female, 9/8), fulfilling the CASPAR criteria [15] with a mean disease duration 4.0 ± 3.6 years and peripheral joint involvement of at least two metacarpophalangeal (MCP) joints and dactylitis of at least one finger were prospectively recruited for the “Analysis of the DActylic Melange” (ADAM) research initiative [16]. At baseline, all patients received methotrexate (MTX) monotherapy. After a baseline MRI scan, they were escalated to Etanercept (Enbrel^®^ 50 mg s.c.) fortnightly and thereafter received a combination therapy of MTX and Etanercept. Follow-up data, including the follow-up MRI scan and clinical and laboratory tests, were available in 13 patients (mean age, 57.0 ± 9.0 years; range, 42–73 years; male/female, 7/6) at 6.2 ± 0.9 months (range 5–8 months) after escalation of treatment. Four of the initial 17 patients had to be excluded from the study prior to the follow-up MRI scan, because they had moved away. In the entire cohort, the Disease Activity Score 28 (DAS 28) [17] was 2.42 ± 0.72 (range, 1.8–4.3; median, 2.2) at baseline and 2.06 ± 0.27 (range, 1.6–2.5; median, 2.1). C-reactive protein (CRP) levels were 0.87 ± 1.35 mg/dL (range, 0.1–5.8 mg/dL; median, 0.3 mg/dL) at baseline and 0.43 ± 0.27 mg/dL (range, 0.1–1.1 mg/dL; median, 0.4 mg/dL) at follow-up. Patient recruitment took place at the Department of Rheumatology from June 2015 until January 2017. The study was approved by the local ethics committee (Medical Faculty, University Dusseldorf, 4962R, date of approval: 1 April 2015). Written and oral informed consent was obtained from all patients before the initiation of the study.

### 2.2. MRI

Baseline (T0) and follow-up (T1) MR imaging of the clinically dominant hand was performed using a 3T MRI scanner (Magnetom Skyra, Siemens Healthineers, Erlangen, Germany) and a dedicated 16-channel receive-only hand coil (3T Tim Coil, Siemens Healthineers) as previously published by our department [16]. Patients were imaged in the prone position with their arms extended overhead and palms facing down (“superman” position).

The imaging protocol was implemented in accordance with the recommendations of the OMERACT working group and included pre- and post-contrast (0.4 mL/kg body weight gadoteric acid [Gd-DOTA], Dotarem, Guerbet Villepinte, France) T1-weighted and non-contrast fat-saturated T2-weighted or short tau inversion recovery (STIR) sequences in two different orthogonal planes. 

Detailed sequence parameters are given in Table 1.

### 2.3. Image Analysis

MR images were independently read and analyzed by two musculoskeletal radiologists (DBA and CS with 3 and 8 years of experience) according to the OMERACT PsAMRIS guidelines [13,14]. The readers were blinded to patients’ and treatment data. Baseline and follow-up scans were independently evaluated in random order. In the case of different scores, cases were discussed by both readers with the assisting opinion of a third reader (PS, trained in musculoskeletal imaging with 8 years of experience) until consensus was reached. Images were scored according to the OMERACT PsAMRIS guidelines [13]. They were evaluated for synovitis (score, 0–3), flexor tenosynovitis (score, 0–3), periarticular inflammation (score, 0 or 1), bone edema (score, 0–3), bone erosion (score, 0–10), and bone proliferation (score, 0 or 1) for the metacarpophalangeal (MCP), proximal and distal interphalangeal (PIP and DIP) joints of digits 2–5. In all joints, the proximal and distal or the dorsal and palmar aspects of the joint were analyzed separately for the presence of bone edema, bone erosions and periarticular inflammation. Scoring was repeated applying a simplified version of PsAMRIS (sPsAMRIS) by the same raters. The time needed to complete the scoring of PsAMRIS and sPsAMRIS per MRI study was recorded throughout the study and comparatively evaluated using Student’s *t*-test.

### 2.4. Development of a Simplified Psoriatic Arthritis MRI Score (sPsAMRIS) and Statistical Analysis

For the development of a simplified scoring system, sPsAMRIS, we applied a single-site weighted summation approach. Priority was assigned to the joints with the highest standardized response mean (SRM) for the change of overall PsAMRIS at baseline (t0) versus follow up (t1). The SRM is an effect size index commonly used to assess a score’s responsiveness to change.

All statistical analyses were performed using The R Project for Statistical Computing, a dedicated software environment for this purpose (version 3.5.1 “feather spray”, the R foundation, https://www.R-project.org).

For descriptive analysis, means, standard deviations, minima, and maxima were determined. The sensitivity for change and their responsiveness was calculated by division of the mean score change by the standard deviation of the change [18]. For PsAMRIS and sPsAMRIS as well as each sub-score, SRM was calculated based on the following Equation (1):

SRM = (mean score t0 − mean score t1)/(SD mean score t0 − mean score t1).(1)

SRMs were defined according to Middel and van Sonderen [19] as large, moderate, small, and trivial responsiveness to therapy based on SRM values of SRM ≥ 0.8, 0.8 > SRM ≥ 0.5, 0.5 > SRM ≥ 0.2, and SRM < 0.2.

Relative efficacy (RE) was calculated for sPsAMRIS compared to PsAMRIS as reference using the following Equation (2):

RE = ((sPsAMRIS SRM)/(PsAMRIS SRM))2(2)

Confidence bounds for RE were estimated by the bootstrap method (based on B = 5000 bootstraps with replacement) and application of the percentile method [20]. RE values > 1 indicate that sPsAMRIS is more efficient than PsAMRIS in detecting change, while RE values < 1 indicate the opposite. For correlation analyses, Pearson’s product-moment correlation with Pearson’s correlation coefficient, r, was determined. Correlation strength was stratified as small (0.1 ≤ r < 0.3), medium (0.3 ≤r < 0.5), and large (0.5 ≤ r < 1) according to Cohen [21]. 

*p*-values of *p* < 0.05 were considered significant. Inter- and intra-rater reliability was calculated by two-way mixed intraclass correlation coefficients, i.e., single-measure ICC (sICC) for intra-rater and average-measure ICC (aICC) for inter-rater reliability based on the initial findings of the two readers before consensus was reached.

## 3. Results

### 3.1. Simplified Score: sPsAMRIS

**Figure 1:**
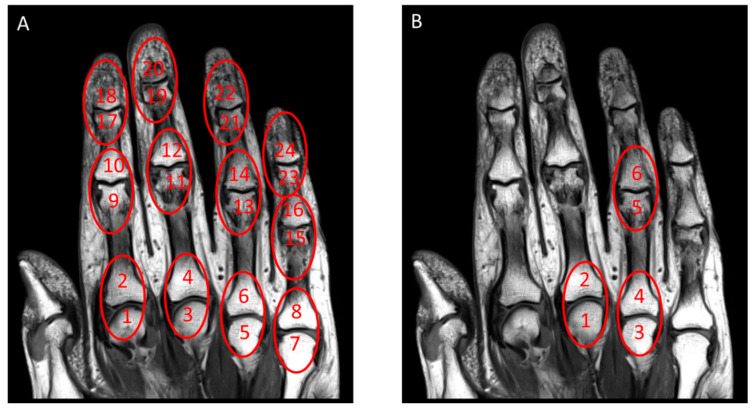
Joint regions and sub-regions in Psoriatic Arthritis Magnetic Resonance Imaging Score (PsAMRIS) versus simplified (s) PsAMRIS. Coronal T1-weighted MR image of a representative patient suffering from psoriatic arthritis (PsA). (**A**) In the full PsAMRIS, inflammatory and destructive changes associated with PsA are assessed in distinct regions and subregions of the hand. Circles indicate scored joints (i.e., regions), while numbers indicate joint sites (i.e., subregions). (**B**) Simplified (s) PsAMRIS. In A, 24 joint sites and/or 12 joints were evaluated, while in B, this number is reduced to 3 joints and/or 6 joint sites that were determined to be most responsive to clinical change.

Changes of overall PsAMRIS and each PsAMRIS sub-score between baseline and follow-up in terms of SRM are summarized in Table 2. For overall PsAMRIS, the MCP joints of digits 3 and 4 and the PIP joint of digit 4 showed the highest SRM values of 0.07, −0.07, and −0.07, respectively, and were hence combined to give the new simplified score sPsAMRIS. The topographical distribution of the regions and subregions to be included in sPsAMRIS as compared to PsAMRIS is detailed in Figure 1.

### 3.2. PsAMRIS and sPsAMRIS during Therapy

**Figure 2:**
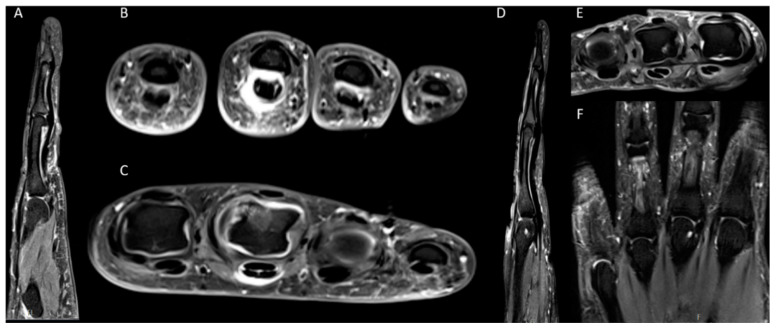
Representative MRI findings in a 57-year-old (**A**–**C**) and a 43-year-old (**D**–**F**) patient suffering from peripheral PsA. (**A**) and (**D**) Sagittal proton density-weighted (PD) fat-saturated (fs) image of the fourth (**A**) and third (**D**) digit (**B**,**C**,**E**) Transversal T1-weighted (T1w) fs image after contrast agent application through the level of proximal interphalangeal (PIP) joints of digits 2–5 (**B**), metacarpophalangeal (MCP) joints of digits 2–5 (**D**) and digits 2–4 (**E**). (**F**) Coronal short tau inversion recovery (STIR) sequence of the MCP joints of the digits 2–5 and the PIP joints of the digits 3 and 4. (**A**,**B**) show distinct periarticular inflammation and flexor tenosynovitis at the PIP joints of digits 3 and 4 and less distinct of the seconds digit. (**C**) illustrates synovitis, flexor tenosynovitis, bone marrow edema and a subtle erosion of the MCP joint of the third digit and moderate periarticular inflammation at the dorsal portion of the MCP joint of the fourth and at the palmar portion of the fifth digit. D-F depict a distinct bone erosion at the MCP joint of the third digit and periarticular inflammation at the MCP joints of the digits 2–4.

The PsAMRIS and sPsAMRIS scores and sub-scores at baseline (i.e., under MTX therapy) and at follow-up (i.e., after escalation to etanercept), are summarized in Table 3. Synovitis, flexor tenosynovitis, and periarticular inflammation were frequently observed in our patient cohort, which is thus reflected in both PsAMRIS and sPsAMRIS (Figure 2). Bone edema and bone erosions, on the other hand were less frequently seen, whereas bone proliferations were rarely detected.

At follow-up, overall PsAMRIS and sPsAMRIS values alongside the sub-scores for synovitis, periarticular inflammation, and bone erosions, were increased as compared to baseline, however, non-significantly. The sub-scores for flexor tenosynovitis and bone edema, on the other hand, were slightly decreased, again non-significantly, at follow-up.

### 3.3. PsAMRIS and sPsAMRIS Sensitivity to Change (Responsiveness) in Terms of Standardized Response Means (SRMs)

The sensitivity to change of PsAMRIS and sPsAMRIS as assessed by the SRMs [22] is summarized in Table 4. Overall, there is mostly trivial sensitivity to change for both PsAMRIS and sPsAMRIS, whereas for the latter, we determined slightly higher absolute SRMs indicating higher sensitivity to change. Only for the sub-scores periarticular inflammation and bone erosion (PsAMRIS) as well as synovitis (sPsAMRIS) did we find slightly higher, yet still low, sensitivity to change (Table 4).

### 3.4. RE and Degrees of Agreement

RE for sPsAMRIS as compared to PsAMRIS was calculated as 29.46 (confidence bounds 2.5/97.5%: 0.00/59.88). Intra- and inter-rater reliability for both scoring systems was high (aICC = 0.95, sICC = 0.92).

### 3.5. Correlation of PsAMRIS and sPsAMRIS

Details of the correlations of PsAMRIS with sPsAMRIS are shown in Table 5. Strong significant correlations between both scores were found for the overall values at baseline and follow-up (baseline, r = 0.75, *p* < 0.01; follow-up, 0.64, *p* < 0.05) as well as for the majority of sub-scores. 

### 3.6. Comparative Analysis of Time Burden

**Figure 3:**
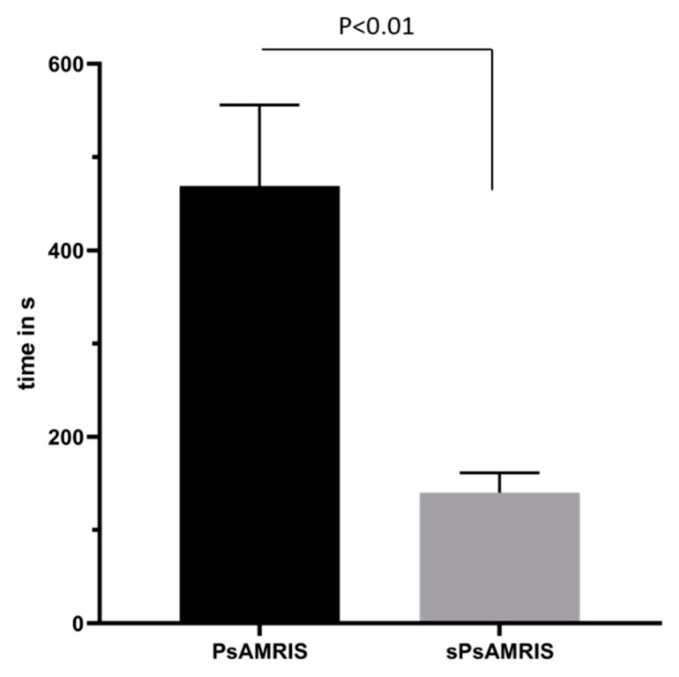
Mean time burden for completion of scoring based on PsAMRIS and sPsAMRIS per MRI study. Scoring on the basis of sPsAMRIS was completed in significantly less time than scoring on the basis of PsAMRIS (*p* < 0.01).

Overall, the time needed for scoring was variable as a function of the number of lesions detected. Per MRI study, significantly higher time burden was recorded for PsAMRIS (mean ± standard deviation, 469 ± 87.03 s; median, 428 s; range, 300–607 s) than for sPsAMRIS (140.1 ± 21.25 s; median, 141 s; 95–174 s) (*p* < 0.001) (Figure 3).

## 4. Discussion

The most important finding of the present study is that sPsAMRIS is a potent diagnostic tool to quantitatively assess and monitor therapy in PsA because of its excellent reliability, higher responsiveness to change, and time efficiency as compared to PsAMRIS. 

In 2009, the OMERACT working group established the PsAMRIS [13] for detecting and grading PsA-related findings. The PsAMRIS is increasingly used for structured semi-quantitative evaluation of peripheral joint changes related to PsA [22,23,24]. Even though PsAMRIS is a sensitive and validated tool for detecting early PsA-related changes [14,25,26], it is of limited use in clinical practice and, hence, primarily used in research, not least because of the substantial time burden. Up to now, however, there has been no alternative to the OMERACT PsAMRIS for semi-quantitative evaluation of joint changes. Therefore, some authors have developed and applied abbreviated versions of PsAMRIS in the context of their research. Feletar et al. scored osteitis, tenosynovitis, and synovitis, without demonstrating correlation with regular PsAMRIS [27]. Our group previously demonstrated that an abbreviated version of the OMERACT Rheumatoid Arthritis Magnetic Resonance Imaging Score (RAMRIS), RAMRIS-5, is a time- and resource-saving alternative to the original version [28]. Similar to our approach for the RAMRIS-5, we reduced the current PsAMRIS to a simplified and abbreviated version that scores 36 instead of 144 items in 3 instead of 12 joints. We found a strong correlation between sPsAMRIS and PsAMRIS at baseline and follow-up after six months of etanercept therapy. Further, sPsAMRIS indicated a very high RE compared to PsAMRIS, which is well in line with other studies [29] and, thus, can be considered to be sufficiently sensitive to change. Additionally, sPsAMRIS significantly decreased the time burden associated with scoring as compared to the regular PsAMRIS. Hence, sPsAMRIS may be a time- and resource-saving alternative for semi-quantitative scoring of PsA-related joint changes of the hand, in particular when screening of large numbers of MRI studies is performed. As PsAMRIS is of limited clinical use due to its time burden, sPsAMRIS is better applicable in clinical and distinct research settings, for example when screening and stratifying potentially eligible patients in disease modifying drug trials. For this purpose, excellent reliability, similar responsiveness to change compared to standard PsAMRIS, and time efficiency have been demonstrated for sPsAMRIS, rendering this scoring system a potential tool in clinical research and clinical work.

Nonetheless, we do not intend to fully replace PsAMRIS by sPsAMRIS because its wide-spread application in clinical research.

Following Ostergaard et al. and Glinatsi et al., who stated potential difficulties in scoring especially DIP and, to a lesser extent, PIP joints, due to a lack of spatial resolution, there are additional arguments for an abbreviation with focus on the MCP joint regions [13,14]. Using a clinical MRI scanner with high field strength of 3 T and a dedicated 16-channel hand coil, we managed to improve spatial resolution considerably, making analysis of the PIP region more accurate. Yet, accurate assessment of the sub-millimeter thin cartilage layers of the PIP and DIP joints remains challenging because of inherent limitations in terms of spatial resolution, signal-to-noise ratio, and partial volume effects [30]. With dedicated wrist coils, let alone specialized high-resolution coils for finger joints, not widely available in radiology departments and clinical scanning of hands oftentimes performed on 1.5 T MRI scanners, the focus on the evaluation of the MCP and PIP joints (as in sPsAMRIS) may strengthen the score’s clinical applicability, validity, and reliability.

Our study has limitations. Since PsA is a disease with several clinical and radiological manifestations, this study focused more on a well-defined and homogeneous patient collective. However, due to our small patient collective included in this exploratory study, our results must be considered preliminary. Further investigations using larger and more varied patient cohorts are required to corroborate our findings and the applicability of sPsAMRIS. Additionally, sPsAMRIS is a data-driven and weighted approach that is derived from this well-defined patient collective, which limits general transferability to other PsA collectives. Since PsA is a very heterogeneous and complex disease, a “one-fits-all” scoring system that is both sensitive and time saving may be even more difficult to establish compared to RA, which tends to be a more homogeneous disease entity.

## 5. Conclusions

The simplified MRI scoring system for PsA-related changes in hands, sPsAMRIS, is a reliable and time-efficient diagnostic scoring tool that is strongly correlated with standard PsAMRIS. Due to its similar responsiveness to change compared to regular PsAMRIS, sPsAMRIS may potentially be used for quantitative assessment and therapy monitoring in PsA. Its clinical applicability beyond our patient collective needs to be demonstrated in larger future study populations. 

## Figures and Tables

**Table 1:** Detailed Magnetic Resonance Imaging (MRI) Sequence Parameters.

Sequence	Orientation	TR/TE (ms)	Flip Angle (°)	Slice Thickness (mm)	FoV (mm × mm)	Aqcuisition Matrix (pixels)	Pixel Size (mm/pixel)
T1w TSE	Coronal	862/27	150	2.5	140 × 140	512 × 512	0.27 × 0.27
T1w TSE + contrast	Coronal	862/27	150	2.5	140 × 140	512 × 512	0.27 × 0.27
STIR	Coronal	5560/31	120	2.5	140 × 140	448 × 314	0.31 × 0.41
T2w TSE fs	Transversal	5694	89	3.0	160 × 160	512 × 358	0.31 × 0.45
PD TSE fs	Sagittal	3150/47	150	2.5	150 × 150	448 × 182	0.33 × 0.82
T1 TSE fs + contrast	Transversal	807/16	90	2.5	130 × 130	384 × 288	0.31 × 0.42

Imaging plane, echo and repetition time (TE/TR), flip angle, slice thickness, field of view (FoV), pixel size, and number of slices are given for all sequences (Short Tau Inversion Recovery, T2-weighted fat-saturated turbo spin echo (T2w TSE fs), T1w TSE, Proton Density TSE fs (PD).

**Table 2:** Changes between T0 and T1 for the overall psoriatic arthritis resonance imaging score (PsAMRIS) and its sub-scores assessed by standardized response means (SRM) (SRM = $\frac{\left(mean\;score\;T0\;-\;mean\;score\;T1\right)}{\left(SD\;mean\;score\;T0\;-\;mean\;score\;T1\right)}$) at the metacarpophalangeal (MCP), proximal interphalangeal (PIP) and distal interphalangeal (DIP) joints of digits 2–5 in a clinical cohort of PsA patients. NaN—not available due to absence of the feature in the study population.

PsAMRIS Sub-Score	MCP				PIP				DIP			
	2	3	4	5	2	3	4	5	2	3	4	5
Overall	−0.01	0.07	−0.07	−0.03	−0.01	0	−0.07	0.00	0	−0.01	0	−0.03
Synovitis	−0.02	0.05	−0.07	−0.04	0.02	−0.03	−0.11	−0.03	−0.05	−0.05	−0.04	−0.04
Flexor tenosynovitis	0.05	0.09	0	0.01	0.01	0.04	−0.05	−0.08	0.04	0.1	0.04	−0.02
Bone Proliferation	−0.01	NaN	−0.01	NaN	−0.01	−0.01	−0.01	−0.01	−0.01	−0.01	0.03	NaN
Periarticular inflammation	−0.03	0.01	−0.01	0	−0.06	−0.05	−0.13	0.00	−0.03	0.01	−0.01	−0.06
Bone edema	−0.02	0.05	−0.07	−0.04	0.02	−0.03	−0.11	−0.03	−0.05	−0.05	−0.04	−0.04
Bone erosion	−0.04	0	−0.04	−0.05	−0.02	−0.01	0.03	0.03	0	−0.04	0.01	0

**Table 3:** Descriptive analysis of PsAMRIS and short (s) PsAMRIS at baseline and at follow-up regarding the overall scores and each sub-score. For each values the mean ± standard deviation (SD), the median and range are presented.

PsAMRIS Sub-Score	Baseline PsAMRIS/sPsAMRIS				Follow-Up PsAMRIS/sPsAMRIS				*p*-Value
	Mean	SD	Range	Median	Mean	SD	Range	Median	
Overall	65.4 /16.3	±17.4 /±4.4	37–93 /9–25	64.0 /15.0	67.5/ 17.2	±14.4 /±3.1	49–98 /14–25	68.0 /16.5	0.958 /0.436
Synovitis	22.1 /6.4	±5.7 /±1.5	13–33 /5–9	22.0 /6.0	24.0 /6.9	±4.7 /±1.3	17–32 /5–9	23.5 /7.0	0.689 /0.149
Flexor tenosynovitis	10.5 /2.9	±5 /±1	3–22 /2–5	10.0 /3.0	9.6 /2.8	±3.1 /±0.7	5–16 /2–4	10.0 /3.0	0.592 /0.602
Bone Proliferation	1.1 /0.2	±1.4 /±0.5	0–4 /0–2	1.0 /0.0	1.1 /0.2	±1.4 /±0.6	0–4 /0–2	1.0 /0.0	1 /0.956
Periarticular inflammation	17.7 /4.5	±3.1 /±1.4	10–22 /1–6	18.0 /5.0	18.9 /4.9	±3.7 /±0.8	9–23 /4–6	19.5 /5.0	0.299 /0.055
Bone edema	6.6 /0.5	±5.5 /±1.2	1–20 /0–4	5.0 /0.0	5.6 /0.4	±6. /±0.9	0–23 /0–3	4.0 /0.0	0.449 /0.336
Bone erosion	7.5 /1.8	±5.5 /±1.6	1–20 /0–5	6.0 /1.0	8.2 /1.9	±5.6 /±1.3	1–19 /0–5	7.0 /1.5	0.316 /0.637

**Table 4:** Standardized Response Means of PsAMRIS and sPsAMRIS and Their Sub-Scores. Sensitivity to change, i.e., responsiveness, of PsAMRIS, sPsAMRIS, and their sub-scores between baseline and follow-up as assessed by SRMs. NaN—not available due to absence of the feature in the study population.

PsAMRIS Sub-Score	PsAMRIS	sPsAMRIS
Overall	−0.02	−0.13
Synovitis	−0.11	−0.21
Flexor tenosynovitis	0.15	0.08
Bone Proliferation	NaN	NaN
Periarticular inflammation	−0.31	−0.16
Bone edema	0.2	−0.13
Bone erosion	−0.29	0.12

**Table 5:** Pearson’s correlation coefficients r of PsAMRIS and sPsAMRIS for the overall score as well as each sub-score at baseline and follow-up. *p*-values of *p* < 0.05 were considered significant and are given in **bold** type.

PsAMRIS Sub-Score	Baseline			Follow-Up		
	Correlation Coefficient *r*	95% Confidence Interval	*p*-Value	Correlation Coefficient *r*	95 % Confidence Interval	*p*-Value
Overall	0.75	0.42; 0.90	**<0.001**	0.64	0.17; 0.87	**0.013**
Synovitis	0.84	0.61; 0.94	**<0.001**	0.74	0.34; 0.91	**0.002**
Flexor tenosynovitis	0.72	0.36; 0.89	**0.001**	0.59	0.09; 0.85	**0.025**
Bone proliferation	0.66	0.27; 0.87	**0.004**	0.66	0.23; 0.88	**0.007**
Periarticular inflammation	0.79	0.50; 0.92	**<0.001**	0.35	−0.22; 0.74	0.223
Bone edema	0.31	−0.2; 0.69	0.221	0.85	0.58; 0.95	**<0.001**
Bone erosion	0.8	0.51; 0.92	**<0.001**	0.74	0.35; 0.91	**0.002**
